# Association of cardiac autonomic neuropathy with arterial stiffness in type 2 diabetes mellitus patients

**DOI:** 10.1186/2251-6581-12-55

**Published:** 2013-12-20

**Authors:** Ataollah Bagherzadeh, Afshin Nejati-Afkham, Yaser Tajallizade-Khoob, Akbar Shafiee, Farshad Sharifi, Morteza Abdar Esfahani, Zohre Badamchizade, Sudabeh Alatab, Hossein Fakhrzadeh

**Affiliations:** 1 Department of Internal Medicine, Cardiology & Cardiac Electrophysiology, Dr Shariati hospital, Tehran University of Medical Sciences, Tehran, Iran; 2 Department of Cardiology, Dr Shariati Hospital, Tehran University of Medical Sciences, Tehran, Iran; 3 Elderly Health Research Center, Endocrinology and Metabolism population Sciences Institute, Tehran University of Medical Sciences, North Karegar Avenue, Dr Shariati Hospital, 5th floor, Tehran, 1411413137 Iran; 4 Department of Cardiovascular Research, Tehran Heart Center, Tehran University of Medical Sciences, Tehran, Iran; 5 Cardiology Department, St alzahr hospital, Isfahan University of Medical Sciences, Isfahan, Iran; 6 Endocrinology and Metabolism Research Center, Endocrinology and Metabolism Research Institute, Tehran University of Medical Sciences, Tehran, Iran

**Keywords:** Heart rate variability, Arterial stiffness, Pulse wave velocity, Diabetes mellitus type 2, Cardiac autonomic neuropathy

## Abstract

**Background:**

Diabetic patients are at the risk of cardiac autonomic neuropathy (CAN) and arterial stiffness. This study aimed to investigate the association of heart rate variability (HRV) as an index for CAN and pulse wave velocity (PWV) as an index for arterial stiffness.

**Methods:**

Uncomplicated diabetes type-2 patients who had no apparent history of cardiovascular condition underwent HRV and PWV measurements and the results were compared with the control group consisting of non-diabetic peers. Also, the findings were adjusted for the cardiovascular risk factors and other confounding factors.

**Results:**

A total of 64 diabetic patients (age= 52.08±8.50 years; males=33 [51.6%]) were compared with 57 controls (age= 48.74±6.18 years; males=25 [43.9%]) in this study. Hypertension, dyslipidemia, and thereby systolic blood pressure and statin use were significantly more frequent in the diabetic group, while the serum levels of cholesterol, HDL-C and LDL-C were significantly higher in the controls. Pulse wave was significantly increased in the diabetic patients (p<0.001). Main HRV parameters were significantly lower in diabetics than in controls. After adjustment for the confounders, PWV and HRV remained significantly different between the groups (p=0.01 and p=0.004, respectively). Multiple logistic regression of the association between pulse wave velocity and HRV index was independently significant both in diabetics and controls.

**Conclusions:**

There exists a significant relationship between heart rate variability and arterial stiffness as a measure for atherosclerosis in diabetic patients, although the role of the confounding factors is noteworthy.

## Introduction

More than half of the mortality in diabetic patients occurs due to cardiovascular disease [1]. Although most of this risk stems from atherosclerosis along with vascular ageing, there are other conditions involved [2]. Cardiac autonomic neuropathy (CAN) is a serious complication of diabetes mellitus, shown to influence both mortality and cardiovascular events in these patients [3]. As the heart rate and vascular tone are regulated via the autonomic system, CAN can increase heart rate variability and decrease myocardial perfusion [4].

On the other hand, heart rate variability (HRV) can be assessed easily to measure CAN, much earlier than its clinical appearance [5]. It has been shown that low HRV is associated with increased mortality in patients with ischemic heart disease or diabetes mellitus [3, 6]. HRV is also a sensitive indicator of baroreflex control, specifically the vagal control [7, 8]. Therefore, arterial stiffness may affect baroreceptor function and thereby, HRV. Increased arterial stiffness evaluated by pulse wave velocity (PWV) and/or augmentation index has been associated with the presence of coronary atherosclerosis and worse cardiovascular prognosis both in general population [9, 10] and specific disease groups, including diabetes mellitus [11]. Therefore, understanding the exact relationship of heart rate variability, as an index for CAN, with arterial stiffness is crucial.

The aims of the present study were to investigate (i) the association of disturbed PWV, as an index for increased arterial stiffness, and HRV, as an index for CAN, in the presence or absence of uncomplicated diabetes mellitus and (ii) the relation of PWV with HRV in uncomplicated diabetic patients.

## Methods

In this case–control study, uncomplicated diabetic type 2 (T2DM) patients who were referred to the Diabetes clinic of Dr. Shariati hospital, Tehran University of Medical Sciences, Tehran, Iran, between August 2011 and December 2012 were recruited. The control group consisted of their spouse or relatives who were proved to be non-diabetic. The inclusion criteria were defined as both sexes, aged between 30 and 65 years. Individuals with clinically proven coronary heart disease, diabetic foot, diabetic retinopathy or nephropathy, renal failure (GFR < 90), history of malignancy, and cirrhosis were excluded from the study. Height was measured with a Stadiometer, and weight was assessed by a calibrated beam balance. Body mass index (BMI) was calculated as weight (Kg) divided by height (M) squared. Blood pressure was measured twice (5 minutes apart) using a standard calibrated mercury sphygmomanometer on both right and left arms after the participants had been sitting calm for at least 10 minutes. The highest blood pressure of two sides was considered as participant’s blood pressure.

All the participants signed an informed consent at the time of recruitment and the study protocol was approved by the ethics committee of Tehran University of Medical Sciences, and the board of research at Dr. Sahriati hospoital.

Diabetes mellitus was defined as patients with fasting blood sugar (FBS) ≥ 126 mg/dL, or 2-h postprandial glucose ≥200 mg/dL or those who were using insulin or oral hypoglycemic agents. Hypertensive patients were those with systolic blood pressure of ≥than 140 or diastolic blood pressure of ≥90 mmHg or were using antihypertensive drugs.

Venous blood samples were drawn from the anticubital vein in the morning after 12 h fasting. The blood samples were centrifuged, and then serum was collected for measuring the biochemical parameters.

In all the participants, FBS and 2-h postprandial glucose levels were measured. Plasma levels of glucose, triglyceride (TG), total cholesterol, high density lipoprotein cholesterol (HDL-C), low density lipoprotein cholesterol (LDL-C), creatinine, and blood urea nitrogen (BUN) were measured by a colorimetric method using Pars Azmoon® kit with an autoanalyzer (Hitachi 902, Boehringer Mannheim Germany). HbA_**1**_C was detected by High-performance liquid chromatography (HPLC) (Knauer, Germany), coupled with fluorescence detector. The method was validated over a linearity range of 1–100 μmol/L of the plasma. The intra- and interassay coefficients of variation (CVs) for all of the measurements were <4%, which was less than allowed CVs.

Evaluation of HRV was performed in a quiet and temperature-controlled room according to the guidelines of the Task Force for Pacing and Electrophysiology [5]. Participants were advised to abstain from caffeinated food and beverages on the day of assessment. Repeat assessments were performed at precisely the same time of day after 48 hours. After 15 minutes of supine rest with a regular and calm breathing pattern, a continuous 10-minute ECG recording was collected using an applanation tonometer interface with HRV software (SphygmoCor, AtCor Medical Pty, Sydney, Australia). The high-frequency (HF band: 0.15–0.45 Hz), low-frequency (LF band: 0.04– 0.15 Hz), and very-low-frequency (VLF band: 0.01–0.04 Hz) components of HRV (measured in absolute units; i.e., ms^**2**^) were obtained. Total power (TP) of HRV was also calculated to be used in regression analysis as a global marker of cardiac autonomic function.

Normalized HF and LF powers were determined by dividing their absolute powers by the total power minus the VLF component and multiplied by 100 [5, 12].

From the electrocardiographic recording, the following statistical and geometric time domain indices were calculated from RR intervals: standard deviation of the NN intervals (SDNN), and the square root of the mean squared difference of successive NNs (RMSSD). Frequency domain variables including total, HF, and LF powers and LF:HF ratio were derived from spectral analysis of successive R-R intervals [5].

HRV measurement was performed after Valsalva and standing maneuvers in addition to supine state, using SphygmoCor software (SphygmoCor, AtCor Medical Pty, Sydney, Australia). For Valsalva maneuver, the participant was requested to blow into the mouthpiece of the device manometer to a pressure of 40 mmHg for 15 seconds. Then the Valsalva ratio was calculated as the relationship between the longest and shortest R-R intervals after strain. For the standing maneuver, the participant was requested to breathe at a normal pace for 5 minutes in the supine state. Then, the participant was asked to change position from supine to a full upright and remain erected until the end of the test. The standing ratio was calculated as longest R-R interval around the 30th beat after standing up to the shortest R-R interval around the 15th beat during standing.

PWV was measured using the SphygmoCor System (AtCor Medical Pty Ltd Head Office, West Ryde, Australia), with the individual in the supine position. The pulse waves of the carotid and femoral arteries were analyzed, estimating the delay according to the ECG wave and calculating PWV. PWV was calculated as the ratio of the distance travelled (calculated as distance in mm of distal minus proximal, where measurements are performed from the supra-sternal notch to the sampling site) and the foot-to-foot time delay between the pulse waves and expressed in meters per second (m/sec).

### Statistical analysis

Continuous data are presented as mean ± SD. For comparing data with normal distribution, unpaired t-test was used. Correlation of variables was demonstrated using Pearson’s and Spearman’s correlation coefficients in normal distributed parametric and nonparametric variables, respectively. For assessment of the association of variables, linear regression and logistic regression were used for parametric and binary variables, respectively. P-values were always 2-sided, and P < 0.05 was considered significant. The SPSS statistical software package (version 18.0 for Windows; SPSS Inc. Chicago, IL) was used for data analysis.

## Results

A total of 64 diabetic patients (age= 52.08±8.50 years; males=33 [51.6%]) were compared with 57 controls (age= 48.74±6.18 years; males=25 [43.9%]) in this study. Table 1 depicts the general characteristics and clinical parameters of both groups. Rate of hypertension, dyslipidemia, and thereby systolic blood pressure and statin use were significantly higher in the diabetic group, while the serum levels of cholesterol, HDL-C and LDL-C were significantly higher in the control group.

**Table 1:** **General characteristics of the study population**

Characteristics	Normal (N=57)	Diabetic (N=64)	P-value
Age (years)	48.74 ± 6.18	52.08 ± 8.50	0.15
Sex (male) %	25 (43.9)	33 (51.6)	0.39
BMI (kg/m^2^)	29.12 ± 5.06	27.93 ± 4.46	0.12
Hypertension	4 (7.0)	23 (35.9)	<0.001
Dyslipidemia	9 (15.8)	36 (57.1)	<0.001
Smoking, n (%)	1 (1.8)	8 (12.5)	0.02
Statin use	2 (3.5)	27 (42.9)	<0.001
FBS (mg/dl)	93.84 ± 13.22	162.60 ± 56.32	<0.001
Hb A1c (%)	5.31 ± 0.68	7.95 ± 1.70	<0.001
Cholesterol (mg) (mg/dl)	201.80 ± 31.04	174.12 ± 38.50	<0.001
Triglyceride (mg/dl) (mg/dl)	173.52 ± 99.17	194.08 ± 120.45	0.42
HDL-C (mg/dl)	46.25 ± 10.74	40.34 ± 8.23	0.001
LDL-C (mg/dl)	114.52 ± 22.43	95.12 ± 23.13	<0.001
Creatinine (mg/dl)	0.95 ± 0.13	0.94 ± 0.15	0.62
BUN	12.88 ± 3.19	12.36 ± 4.15	0.45
Hemoglobulin	14.87 ± 4.37	14.30 ± 1.44	0.34
SBP (mmHg)	123.46 ± 14.09	131.00 ± 17.33	0.01
DBP (mmHg)	77.47 ± 10.11	76.03 ± 8.73	0.4

BMI: Body mass index; BUN: Blood urea nitrogen; DBP: Diastolic blood pressure; FBS: Fasting blood sugar; HDL: High density lipoprotein; hs-CRP: High sensitive C-reactive protein; IFG: Impaired Fasting Glucose; LDL: Low density lipoprotein; SBP: Systolic blood pressure.

Table 2 compares the PWV and HRV indices between the study groups. Pulse wave velocity was significantly increased in the diabetic patients (p<0.001). RMSSD and HRV index were significantly lower in diabetics than in controls (p=0.02 and p<0.001, respectively). Significant reduction of total power was also observed in the diabetic patients relative to controls.

**Table 2:** **Comparing pulse wave velocity and heart rate variability indices between the study groups**

Variables	Normal (N=57)	Diabetic (N=64)	P-value
Pulse wave velocity (m/s)	8.00 ± 1.61	10.11 ± 2.45	<0.001
RMSSD (ms)	31.78 ± 21.27	20.13 ± 19.51	0.02
HRV index	7.78 ± 2.94	5.57 ± 2.22	<0.001
Heart rate(bpm)	68.95 ± 9.44	73.64 ± 8.41	0.005
PNN 50	9.58 ± 14.56	3.01 ± 7.10	0.002
LF norm (ms2)	55.98 ± 18.79	61.65 ± 20.99	0.12
HF norm (ms2)	44.02 ± 18.78	38.34 ± 20.99	0.12
LF:HF ratio	1.83 ± 1.70	2.72 ± 2.73	0.03
Total power (ms2)	1133.17 ± 1266.26	703.73 ± 1279.76	0.06
Valsalva ratio	1.58 ± 0.28	1.44 ± 0.31	0.01
Standing ratio	1.27 ± 0.13	1.23 ± 0.20	0.29

HF: High frequency; HRV: Heart rate variability; LF: low frequency; RMSSD: the square root of the mean squared difference.

After adjustment for the confounding variables, including age, BMI, hypertension, dyslipidemia, smoking, statin use, FBS, total cholesterol and systolic blood pressure, PWV and HRV remained significantly different between the groups (p=0.01 and p=0.004, respectively).

As HF power was highly correlated with the total power and their observed associations were similar, we performed our analyses using total power. Unadjusted correlations between total power and cardiovascular parameters showed that the PWV was positively correlated with the total power in diabetic patients while the correlation was reverse in non-diabetic controls and total population. Also, hypertension and triglyceride levels were positively correlated with the total power in the diabetic patients (Table 3).

**Table 3:** **Partial and total correlations of the total power with PWV and other cardiovascular parameters**

Characteristics	Nondiabetic subjects	Diabetic subjects	All subjects
Age (years)	−0.42 †	−0.16	−0.14
Sex (male) %	0.17	0.06	−0.07
BMI (kg/m^2^)	0.07	−0.23	−0.06
Hypertension	0.2	0.28 *	0.15
Dyslipidemia	0.14	0.19	0.2 *
Current smoker (%)	0.02	−0.17	−0.17
Statin use	−0.11	−0.14	−0.12
FBS (mg/dl)	−0.23	−0.003	−0.02
Hb A1c (%)	0.004	−0.09	0.09
Cholesterol (mg) (mg/dl)	−0.04	0.04	−0.03
Triglyceride (mg/dl) (mg/dl)	−0.02	0.25 *	−0.08
HDL-C (mg/dl)	−0.13	−0.02	−0.10
LDL-C (mg/dl)	−0.04	−0.10	−0.001
Creatinine (mg/dl)	−0.10	0.18	0.06
BUN	−0.06	−0.05	−0.12
Hemoglobulin	−0.20	0.21	0.02
SBP (mmHg)	−0.10	−0.31 *	−0.12
DBP (mmHg)	−0.35 †	−0.36 †	−0.32 †
Pulse wave velocity (m/s)	−0.28 *	0.37 †	−0.19 *

* p<0.05.

† p<0.01.

BMI: Body mass index; BUN: Blood urea nitrogen; DBP: Diastolic blood pressure; FBS: Fasting blood sugar; HDL: High density lipoprotein; hs-CRP: High sensitive C-reactive protein; IFG: Impaired Fasting Glucose; LDL: Low density lipoprotein; SBP: Systolic blood pressure.

Multiple logistic regression showed that pulse wave velocity was independently associated with HRV index both in diabetics and controls (Table 4). This relationship showed a dose–response pattern throughout the distribution of HRV. On the other hand, this relation lost its significance after adjustment for diabetes and particularly in the multiple regression model, including age, hypertension, dyslipidemia, smoking, BMI, and systolic blood pressure.

**Table 4:** **Association between pulse wave velocity and quartiles of HRV index in multivariate logistic regression model**

	Unadjusted OR (CI)	Diabetes adjusted OR (CI)	Full adjusted OR (CI)
Fist quartile of HRV index†			
Second quartile of total power	0.73 (0.57, 0.92)	0.81 (0.63, 1.04)	0.93 (0.81, 1.06)
Third quartile of total power	0.51 (0.38, 0.71)	0.57 (0.41, 0.80)	0.55 (0.37, 0.84)
Fourth quartile of total power	0.55 (0.40, 0.74)	0.64 (0.47, 0.89)	0.91 (0.61, 1.37)
P trends	<0.001	0.001	0.01

† First quartile of the HRV was considered as the reference.

CI: confidence interval; HRV: Heart rate variability; OR: Odd’s ratio; PWV: Pulse wave velocity.

## Discussion

In this study, we observed that considerable cardiac autonomic dysfunction exists in the uncomplicated diabetic patients in comparison with the normal controls, as well as an increase in the arterial stiffness, measured by pulse wave velocity.

Current evidence confirms that HRV is a good measure of cardiac autonomic neuropathy in diabetic patients and its decrease is accompanied by increased mortality and morbidity [13]. In our study, increased resting heart rate and decreased Valsalva ratio and standing ratio in the diabetic patients illustrate the parasympathetic involvement of the autonomous system as compared with the normal controls. This has also been shown previously both in type 1 and type 2 diabetes patients [14, 15].

Decreased HRV in the uncomplicated diabetes patients of our study and previous studies highlights the obscure process of autonomic neuropathy in diabetic patients that begins even before clinical atherosclerotic cardiovascular disease becomes apparent [16].

It has also been shown that surrogate atherosclerosis markers were associated with lower HRV, and increased carotid intima media thickness (CIMT) in T2DM participants was significantly associated with decreased HRV, independent from conventional cardiovascular risk factors [16]. Therefore, the presence of cardiac autonomic neuropathy should be considered much earlier in the course of diabetes, rather than after the development of clinical cardiovascular disease.

PWV is known as a potentially applicable atherosclerotic risk marker irrespective of classical cardiovascular risk factors and ethnicity [17]. In previous studies, arterial stiffness assessed by pulse wave analysis had a prognostic value for cardiovascular morbidity and mortality, mostly in hypertensive patients [18–20]. One study demonstrated that increased aortic pulse wave velocity was associated with the presence of angiographic coronary artery disease in overweight and obese patients, although the arterial stiffness indices were not consistently associated with obesity [21]. Similarly, it has been shown that cardiac parasympathetic function is a strong predictor of large arterial stiffness, in young type 1 diabetes patients without macrovascular and renal complications [22]. In the Pittsburgh Epidemiology of Diabetes Complications study, cardiac autonomic neuropathy was associated with increased arterial stiffness indices, in patients with childhood-diagnosed type 1 diabetes [23]. Moreover, a novel relationship between arterial stiffness, hyperinsulinaemia and autonomic neuropathy in a Type 2 diabetic population has been shown in a study which signifies their pathogenic roles in the development of cardiovascular disease in diabetic patients [13].

Based on our findings and previous works, one could suggest that atherosclerosis, both as a result of diabetes and increased age is influenced by the cardiac autonomic neuropathy, which in turn results in increased risk of cardiovascular diseases and related mortality in the type 2 diabetes patients.

### Study limitations

Among limitation to the study, we can mention that our measurements were cross-sectional and we could not assess how the changes in both HRV and PWV through time, as well as the fluctuations in the serum glucose levels and glycosated hemoglobin, affect each other. We also did not perform glucose tolerance test in the normal controls, so there is a probability that those with glucose intolerance may have some degrees of disturbed HRV and PWV. Also the effects of the type of diabetes treatment and other prescribed medications on HRV and PWV need to be investigated in future studies.

## Conclusion

In this study, we observed increased arterial stiffness and decreased heart rate variability in the uncomplicated type 2 diabetes patients as compared with normal controls. The relationship between heart rate variability indices and pulse wave velocity was significant after adjustment for diabetes; however, this effect was lost after adjustment for confounders. Based on the findings of this study, it seems that there exists a relationship between heart rate variability and arterial stiffness as a measure for atherosclerosis in diabetic patients, although the role of the confounding factors should be taken into account.

## Abbreviations

CAN: Cardiac autonomic neuropathy
HRV: Heart rate variability PWV: Pulse wave velocity T2DM: Diabetic type 2
GFR: Glomerular filtaration rate
BMI: Body mass index
FBS: Fasting blood sugar
TG: Triglyceride
HDL-C: High density lipoprotein cholesterol
LDL-C: Low density lipoprotein cholesterol
BUN: Blood urea nitrogen
HPLC: HIGH-performance liquid chromatography
HF: The high-frequency
LF: Low-frequency
VLF: Very-low-frequency
SDNN: Standard deviation of the NN intervals
RMSSD: Square root of the mean squared difference of successive NNs
CIMT: Carotid intima media thickness.
